# The influence of job content and autonomy on psychological well-being through work identity and job crafting among pharmacists in Pakistan

**DOI:** 10.1371/journal.pone.0350128

**Published:** 2026-05-29

**Authors:** Rana Muhammad Zahid Mushtaq, Naeem Rasool, Zaka Ur Rehman, Usama Bin Naeem, Muhammad Azeem, Tehreem Fayyaz, Mehmood Ahmad, Waqas Ahmad

**Affiliations:** 1 Institute for Regeneration and Repair (IRR), College of Medicine and Veterinary Medicine, The University of Edinburgh, Edinburgh, United Kingdom; 2 Al-Shifa Institute of Health Sciences, Narowal, Pakistan; 3 Department of Pharmacy, University of Lahore, Lahore, Pakistan; 4 Department of Pharmacology, Riphah College of Veterinary and Animal Sciences Lahore, Lahore, Pakistan; 5 Department of Allied Health Sciences, The Superior University, Lahore, Pakistan; 6 Department of Pharmacology and Toxicology, Faculty of Veterinary and Animal Sciences, The Islamia University of Bahawalpur, Bahawalpur, Pakistan; 7 Department of Pathology, Faculty of Veterinary and Animal Sciences, University of Veterinary and Animal Sciences Lahore, Lahore, Pakistan; University of Toronto, CANADA

## Abstract

**Background:**

Psychological well-being of pharmacists is necessary for safe and effective healthcare delivery, particularly in high-stress environments. The present study was aimed to investigate the influence of job content on psychological well-being among the registered pharmacists in Pakistan along with the mediating roles of work identity and job crafting.

**Methods:**

Data were collected from 564 licensed pharmacists on standardized instruments for psychological well-being (Ryff’s PWB Scale), job content (Karasek’s JCQ), work identity, and job crafting behaviors. Structural equation modeling using RStudio tested the direct, indirect, and serial mediation effects, with bootstrapped 95% confidence intervals.

**Results:**

Confirmatory factor analysis indicated acceptable model reliability (e.g., Job Crafting α = 0.94, CR = 0.96; PWB α = 0.66, CR = 0.85). Bivariate correlations showed that psychological well-being was moderately associated with job crafting (r = 0.49, p < 0.01) and strongly with work identity (r = 0.57, p < 0.01). SEM analysis showed that Job content had no significant direct effect on well-being (β = −0.0362, p = 0.56), two significant indirect effects were observed: via work identity (β = 0.1397, p < 0.05) and job crafting (β = 0.1295, p < 0.05). The sequential pathway; Job content → work identity → job crafting → psychological well-being also yielded a smaller but significant effect (β = 0.0278, p < 0.05).

**Conclusion:**

Our findings suggest that work identity and job crafting may mediate the relationship between job content and psychological well-being; however, these results should be interpreted cautiously given the model fit limitations.

## Introduction

A person’s entire emotional and mental state is referred to as their psychological well-being, encompassing positive traits such as autonomy, personal growth, a guiding goal or sense of purpose in life, stress management skills and a capacity to build healthy relationships [1,2]. It involves controlling one’s emotions, perceiving clearly, engaging in constructive tasks, having satisfying relationships, and not having mental illness like anxiety or depression [3]. Pharmacists, as healthcare professionals, play a critical role in patient care [4], often working in high-stress environments with demanding responsibilities [5]. Their psychological well-being is vital for their ability to perform their duties effectively and thus needs more attention [6]. Hospital pharmacists have particularly high stakes, as any error they make while managing medication could have serious health effects on their patients [7,8]. The burden of several duties often leads to stress, reduced fulfilment with work and a decline in general well-being [9]. Studies have additionally shown the severe ongoing stress of work-related pressure, anxiety and depression on the onset of hypertension and a marked decline in general quality of life [10]. Hence, overall well-being of pharmacists encompasses physical health, mental stability and work life satisfaction [11,12].

The work organization and the workload of pharmacists, their autonomy in making decisions, and the support of colleagues are important factors that influence their mental health [13]. These dimensions are often defined as job content and demonstrate the degree of perceived autonomy, and the impact of this perception of control on motivation and stress. In recent studies, it has been stressed that the overall psychological well-being of employees is likely to increase when they are provided with a higher degree of decision-making latitude and when they feel supported in the workplace [14]. On the other hand, job pressure, role ambiguity, and lack of autonomy remain typical issues in the healthcare environment and often lead to emotional burnout and disengagement [15,16]. In turn, the analysis of job content and autonomy performed simultaneously provides a more narrow and realistic insight into the factors that influence the well-being of pharmacists in the Pakistani health system that is highly demanding.

Job crafting involves proactive strategies that pharmacists can employ to enhance their work experience and mitigate job stress, thereby improving their psychological well-being [17]. By taking on activities that align with their areas of expertise, such as medication administration, patient counseling or specialist areas like geriatrics or diabetes care, pharmacists can practice task crafting [18]. This method can help them feel more accomplished and less burned out, which lessens their mental exhaustion [19]. Pharmacists can establish enduring relationships with their patients and colleagues by employing rationale crafting, which fosters a productive workplace [20]. Strong interpersonal relationships can lessen stress and feeling of loneliness, while limiting cognitively taxing contacts help maintain mental energy [21,22]. In addition to the structural factors of job content, pharmacists often take proactive steps to create and enhance their work experience, which is called job crafting [23].

Autonomy refers to the level of freedom employees have to make decision about how they perform their tasks., which can empower and inspire them [24]. Workplace autonomy is crucial for pharmacists as it directly effects their performance, mental health and job satisfaction [25]. Being autonomous, pharmacists can decide on their own using their clinical, drug administration and counseling skills [26]. They are more confident and dedicated because they can make decisions about their work [27]. In addition to improving patient outcomes, autonomy allows pharmacists to fully utilize their knowledge, which lessens the annoyance that can be caused by strict procedures and overbearing monitoring [28]. Eventually, pharmacists can succeed professionally and preserve their mental health in a work atmosphere that encourages autonomy [29].

The degree to which employees engage as well as find personal fulfilment in their work is known as work identity, and it is vital for mental health [30,31]. A pharmacist’s work identity is a crucial part of their professional life as it influences how they see themselves and their duties in the healthcare system [32]. When pharmacists can relate to their jobs with ease, they are more likely to feel proud and dedicated, which boosts their feeling of purpose and job satisfaction [33]. Pharmacist’s work identities are frequently shaped by the activities they perform on a daily basis, [34] their relationships with patients and health care team and the acknowledgement they get for their efforts [35,36]. Strong professional identities are associated with pharmacists who take charge of their duties and regard themselves as vital members of the team [37]. Hence, a well-formed work identity supports their psychological well-being, help them navigate challenges and maintain resilience [38,39].

The association between job stress and psychological well-being is grounded in several key theoretical frameworks that investigate how workplace stresses affect mental health and general well-being [40]. The transaction model of stress and coping by Lazarus and Folk-man posits that stress is a result of correlation between an individual and their environment [41]. According to the Conservation of resource theory, stress arises when there is a possibility of resource loss or lack of resource gain after investment [42]. Resources can be physical, social and psychological [43]. Workplace stress can exhaust these resources, which lowers psychological well-being [44]. Hospital pharmacists experience significant job stress due to their demanding duties [45]. Besides having to assess a large number of prescriptions, they must also deal with serious and dangerous repercussions of pharmaceutical errors [46]. As a result, in order to satisfy these requirements, their strategy must be improved. Inadequate resources such as unfavorable working circumstances or little schedule flexibility, can exacerbate stress accumulation and lead to deteriorating health [47].

Empirical studies showed that coping strategies mediate the relationship between a person’s well-being and performance [48]. Recently, a study was carried out in which 782 employees from different industries in Jordan were surveyed to investigate the impact of job crafting, proactive personality and autonomy on psychological well-being. The results showed a positive correlation between these factors and the mental health of the employees [49]. In 2013, a study investigated the connection between 11,700 workers’ physical and mental health and outcomes like job performance ratings, inactivity and intention to stay, both prospectively and cross-sectionally over a period of 12 months. It was seen that well-being remained an accurate indicator of all employee’s outcomes and the improvement in their performance is positively related to healthy mental state [50]. The strategies like work identity, rationale task crafting and decision making power lead to proactive management of responsibilities, increased attention to details and greater commitment to patient care [51]. Pharmacists with better mental health are likely to have better interactions with patients, providing more thorough consultation and advice, which can improve patient outcomes and satisfaction [52].

Pakistani healthcare workers deal with numerous difficult problems like not having enough resources, [53] political and administrative problems and few prospects for professional growth [54]. Increased stress, burnout and discontent with one’s work are caused by these reasons [55]. Their psychological well-being is adversely impacted by the continual strain of working in overcrowded and under-resourced workplaces [56]. Furthermore, people are discouraged from getting help due to the stigma associated with mental health, which exacerbates stress and mental health problems [57]. Increasing job structural resources, decreasing hindering job demands [58], increasing social job resources and decreasing job insecurities are all necessary to meet these issues [59].

**Fig 1:**
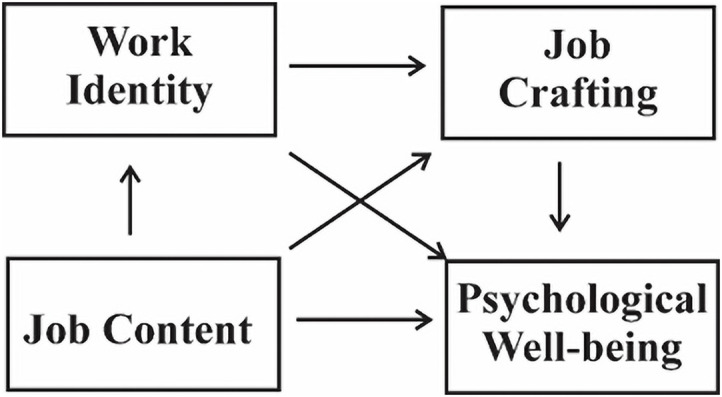
Hypothesis path analysis of the study constructs.

There exists a good amount of study on the topic of mental health and psychological well-being, but not much of it focuses on how job stress affects hospital pharmacist’s well-being, especially in an underdeveloped country like Pakistan. The majority of the existing literature focuses on healthcare professionals, frequently ignoring particular difficulties faced by pharmacists. Furthermore, the possible mitigating impact of flexible work schedules on workplace stress within this potential generation has received less consideration. The present study aims to investigate the understudied issues by clearly evaluating the experiences of hospital pharmacists in Pakistan. Specifically, we hypothesized that: (1) job content would be positively associated with psychological well-being; (2) this relationship would be mediated by pharmacists’ work identity; and (3) job crafting would also mediate the relationship between work identity and psychological well-being. We further hypothesized that the relationship between job content and psychological well-being is serially mediated by work identity and job crafting as Hypothesis 4 (Fig 1).

## Materials and methods

### Study dataset and population cohort

The data were collected from the registered pharmacists present Pakistan during June-November 2024. A comprehensive list was compiled using the pharmacist’s registration records and online databases.

### Sample size quantification

The sample size was calculated using Cochran’s formula for known populations according to the total count of registered pharmacists in Pakistan. The sample size of 557 pharmacists was calculated by assuming a 99% confidence level, 5% margin of error and an anticipated response rate of 70% [60]. In order to reduce the effect of potential non-responsiveness, the target sample size was further increased (n = 597) so that the study sample could be statistically representative of the pharmacist population [61].

### Eligibility criteria

Only pharmacists who had a valid license in Pakistan were allowed to take part in the study. Participants were required to understand the study’s purpose and procedures, provide written informed consent, and should voluntarily agree to take part in the study with the option to discontinue at any stage. We excluded pharmacists with mental health conditions or those in clinical management for psychological disorders to avoid factors that might influence the assessment of their psychological well-being, job crafting, work identity and job characteristics. These criteria were applied to ensure a relatively homogeneous professional sample and to minimize potential confounding influences, which is consistent with similar cross-sectional studies conducted among healthcare professionals.

### Ethical approval

The study was conducted after approval by the Institutional Ethics Review Committee of The Islamia University of Bahawalpur (No: DR/2054 dated. May 2024). Information was given and consent was taken from all participants before data collection and anonymization was ensured for confidentiality and privacy.

### Data collection procedures

Primary data were collected using structured, self-administered questionnaires which were designed to assess key constructs of the study, including job crafting behaviors, work identity, job content characteristics, and psychological well-being among pharmacists. To facilitate the data collection process, the research team both in-person and email-based explanations of the study’s objectives, procedures, and ethical considerations. Data collection was performed through both on-site visits and digital distribution through a secure, password-protected online google forms platform [62]. Trained research assistants visited the eligible pharmacists with a brief overview of the study, obtained their written informed consent, and distributed printed questionnaires to those who preferred a physical format. Completed forms were then collected on-site at a later scheduled time. Participants were given the freedom to choose their preferred method of response (either paper-based or digital) to enhance accessibility and response rates. Engagement in study was completely optional and individuals were made well aware of their right to quit the study at any stage without justification.

### Data cleaning and missing data management

After data collection, the dataset was subjected to cleaning and the responses were initially screened for incomplete entries, and data entry errors. Duplication in the records was identified and removed. Additionally, for the paper-based submissions, the entries were meticulously cross-checked against the original forms during digital transcription to maintain data integrity. Responses that contained more than 10% missing values in core variables, including job crafting, work identity, job content (e.g., decision latitude, job demands), and psychological well-being were excluded from the analysis to avoid compromising the validity of results. For entries with minor missing data in non-essential fields, such as demographic characteristics, mean substitution was used to impute isolated missing values. Notably, no changes were performed for the primary study constructs to preserve the authenticity of the data and to ensure that the dataset used for subsequent statistical analysis was reliable.

### Measurement scales

#### Psychological well-being (Dependent variable).

The psychological well-being of participants was measured using Ryff’s Scales of Psychological Well-Being (PWB) which is a validated multidimensional instrument designed to assess positive functioning and overall mental wellness. The scale covers six core dimensions; autonomy, environmental mastery, personal growth, positive interpersonal relations, purpose in life, and acceptance of one self [63]. Each item is rated on a Likert scale and the higher scores show greater well-being across the respective domains. The instrument has demonstrated strong psychometric properties, including high internal consistency (Cronbach’s alpha typically > 0.80 across subscales) and construct validity. Although we used the Ryff PWB scale but the prior studies have frequently reported marginal CFA fit and item/method effects in non-original contexts [64–66]. These precedents motivated our decision to report both reliability (α, CR, AVE) and multiple fit indices, and to interpret paths cautiously.

#### Work identity (Mediator 1).

Work identity among the participants was assessed using the Work-Related Basic Need Satisfaction and Identity Scale regarding three basic psychological needs such as autonomy, competence, and relatedness within their work environment. The responses are rated on a Likert scale ranging from “Strongly Disagree” to “Strongly Agree” [67].

#### Job crafting (Mediator 2).

In the present study, we measured Job crafting using the Job Crafting Scale developed by Tims et al. [68]. This scale consists of 21 items encompassing four dimensions; increasing structural job resources, increasing social job resources, increasing challenging job demands, and decreasing hindering job demands. The response for each item were quantified on a 5-point Likert scale, i.e., “Strongly Disagree” to “Strongly Agree”.

#### Job content (Independent variable).

**Fig 2:**
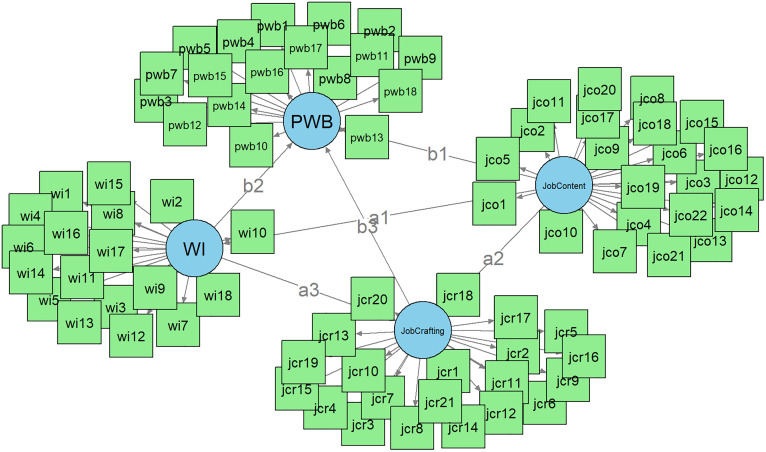
Graphical illustration of the study construct.

To assess the perceptions of participants regarding their job characteristics such as job demands, decision latitude (control), and social support, the Job Content Questionnaire (JCQ) was used in the present study [69]. The JCQ responses were also based on a Likert-type scale ranging from 1 (“Strongly Disagree”) to 4 (“Strongly Agree”). The final construct of latent variable has been summarized in Fig 2.

### Statistical analysis

All statistical analyses were carried out in RStudio using LAVAAN, and PSYCH packages. Prior to the desired analyses, the Kaiser-Meyer-Olkin (KMO) measure of sampling adequacy and Bartlett’s Test of Sphericity were applied to assess the appropriateness of the data for factor analysis. Subsequently, Confirmatory Factor Analysis (CFA) was performed to validate the measurement properties of the latent constructs such as Job Content, Work Identity, Job Crafting, and Psychological Well-Being through checking the internal consistency (Cronbach alpha), average variance extracted (AVE), composite reliability (CR), and standardized factor loadings. Descriptive statistics were calculated for all major variables to evaluate data distribution and to check for normality assumptions. Pearson’s bivariate correlation analysis was used analyzed the associations among the key constructs. To assess the predictive effects of Job Content, Job Crafting, and Work Identity on Psychological Well-Being, a series of multiple regression analyses were conducted. In these models, demographic covariates (e.g., age, gender, education level) were initially included; however, none demonstrated statistically significant relationships with the dependent variable or the mediators and were therefore excluded from the final model for parsimony. Mediation analysis was done to evaluate the direct and indirect effects of Job Content on Psychological Well-Being, operating through Work Identity and Job Crafting both independently and in serial. A bias-corrected bootstrapping technique with 5000 resamples was used to generate 95% confidence intervals for the indirect effects. Mediation was considered as statistically significant if the zero was not present in the confidence interval.

## Results

### Univariate analysis of demographics

**Fig 3:**
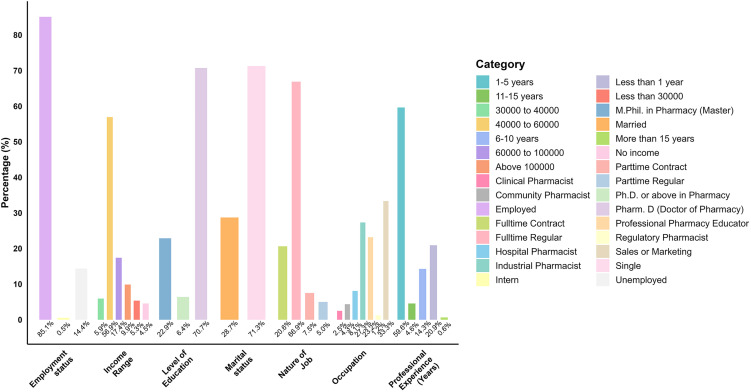
Demographic distribution of hospital pharmacists (*n* = 564). Frequency and percentage distribution of participants by marital status, education level, employment status, job nature, occupation, professional experience, and income range.

The study sample was comprised of 564 participants having a median age of 29.73 years (IQR = 4.78) and the most of the respondents were single (71.28%). Regarding the education level, the majority held a Doctor of Pharmacy (Pharm.D.) degree (70.74%), followed by 22.87% with an M.Phil. in Pharmacy. Almost 85.11% were employed, while 14.36% were either unemployed or interns (0.53%). The most respondents (66.88%) held full-time regular positions of which 33.33% were in sales or marketing, followed by industrial pharmacy (27.33%) and pharmacy education (23.19%). Most participants had been working for 1–5 years (59.63%), while 20.91% had less than a year of experience. Another 14.29% had been in the field for 6–10 years. When it came to income, the majority (56.92%) earned between 40,000–60,000 PKR per month. Others earned either above or below this range, with 4.55% reporting no income at all (Fig 3).

### Model adequacy analysis

The KMO measure showed an acceptable value of 0.862 which suggests the variables shared sufficient common variance for further factor analysis techniques. In addition, Bartlett’s Test of Sphericity produced a highly significant result (χ² = 10,990.745, df = 3081, p < 0.0001), indicating that the correlation matrix is significantly different from an identity matrix (Table 1).

**Table 1:** Kaiser-Meyer-Olkin (KMO) measure of sampling adequacy and Bartlett’s Test of Sphericity for the measurement model.

KMO and Bartlett’s Test		
Kaiser-Meyer-Olkin Measure of Sampling Adequacy.		0.862
Bartlett’s Test of Sphericity	Approx. Chi-Square	10990.745
	df	3081
	Sig.	P < 0.0001

### Confirmatory factor analysis

The internal consistency of the constructs was recorded as within acceptable ranges. Job Crafting demonstrated excellent internal consistency, with a Cronbach’s alpha of 0.940 and a CR of 0.960, exceeding the conventional threshold of 0.70. Its AVE of 0.550 also satisfied the minimum requirement of 0.50. Work Identity showed a Cronbach’s alpha of 0.700 and a CR of 0.840. Similarly, Job Content showed good internal consistency (α = 0.800; CR = 0.900). Psychological Well-Being displayed marginal internal consistency (α = 0.660), with a CR of 0.850, but an AVE of 0.350, which also fell below the acceptable threshold (Table 2). Although some constructs demonstrated AVE values below the recommended threshold of 0.50, they were retained based on acceptable composite reliability (CR > 0.70) and theoretical relevance, as suggested in prior SEM literature. Additionally, several negatively loaded items reflect reverse-coded statements, which were retained after careful verification to preserve the conceptual integrity of the original scales. Therefore, the measurement properties of certain constructs should be interpreted with caution, and the results are considered exploratory.

**Table 2:** Internal consistency (Cronbach’s alpha), composite reliability (CR), and average variance extracted (AVE) for each study construct.

	Cronbach’s Alpha	CR	AVE
Job Crafting	0.940	0.960	0.550
Work Identity	0.700	0.840	0.310
Job Content	0.800	0.900	0.360
Psychological Well-Being	0.660	0.850	0.350

The standardized factor loadings for all items of Job Crafting were positive and generally strong (0.47 to 0.81). While, Work Identity exhibited relatively negative or weak associations with the latent construct (e.g., Item 1: −0.32; Item 3: −0.32; Item 14: −0.53). Similarly, items of Job Content had both positive and negative loadings (Item 3: −0.42; Item 4: −0.46; Item 7: −0.60). The factor loadings for Psychological Well-Being also had slight variations (Item 2: −0.52; Item 9: −0.37; Item 18: −0.42). Overall, Job Crafting exhibited strong psychometric properties but the other three constructs, especially Work Identity and Psychological Well-Being had acceptable factor loadings (Table 3).

**Table 3:** Standardized factor loadings for the items measuring Job Crafting, Work Identity, Job Content, and Psychological Well-Being.

Standardized Factor Loadings				
Items	Job Crafting	Work Identity	Job Content	Psychological Well-Being
1	0.79	−0.32	0.52	0.6
2	0.81	0.48	0.57	−0.52
3	0.77	−0.32	−0.42	0.6
4	0.81	0.44	−0.46	0.6
5	0.77	−0.3	0.61	0.37
6	0.74	0.45	0.73	0.72
7	0.72	−0.35	−0.6	0.77
8	0.58	0.55	0.63	0.71
9	0.71	0.62	0.76	−0.37
10	0.58	−0.36	0.64	0.6
11	0.69	0.71	0.64	−0.34
12	0.65	0.73	0.46	0.54
13	0.64	0.68	0.6	−0.36
14	0.58	−0.53	0.64	0.66
15	0.52	0.56	0.67	−0.31
16	0.66	0.66	0.5	0.67
17	0.71	0.63	0.4	0.68
18	0.7	−0.51	0.53	−0.42
19	0.6	–	0.33	–
20	0.47	–	0.3	–
21	0.67	–	0.37	–
22	–	–	0.3	–

### Model fit analysis

The acceptable thresholds for good model fit are Root Mean Square Error of Approximation (RMSEA) ≤ 0.06, Standardized Root Mean Square Residual (SRMR) ≤ 0.08, Comparative Fit Index (CFI) and Tucker–Lewis Index (TLI) ≥ 0.90, and the ratio of chi-square to degrees of freedom (χ²/df) ≤ 3.0. In our constructs, Job Crafting showed an RMSEA of 0.154, SRMR of 0.086, and a χ²/df ratio of 5.54, while the CFI and TLI values (0.76 and 0.734, respectively). Work Identity and Job Content demonstrated the weak fit statistics among the constructs. Both displayed high RMSEA values (0.178 and 0.180, respectively), elevated SRMR (0.182 and 0.131). Psychological Well-Being had an RMSEA of 0.133 and SRMR of 0.134, respectively and a χ²/df of 4.41 that are indicative of a marginal fit. The overall measurement model exhibited comparatively improved model fit: RMSEA = 0.095, SRMR = 0.105, CFI = 0.578, TLI = 0.566, and χ²/df = 2.75 (Table 4). Although the global model fit indices (CFI and TLI) were below recommended thresholds, the presence of theoretically consistent and statistically significant path coefficients, along with bootstrapped indirect effects, supports cautious interpretation of the hypothesized relationships. Therefore, the findings are interpreted as exploratory rather than confirmatory.

**Table 4:** Model fit indices (RMSEA, SRMR, CFI, TLI, and χ²/df) for each construct and the overall measurement model.

	RMSEA	SRMR	CFI	TLI	χ²/df
Job Crafting	0.154	0.086	0.76	0.734	5.54
Work Identity	0.178	0.182	0.569	0.512	7.08
Job Content	0.18	0.131	0.539	0.491	7.24
Psychological Well-Being	0.133	0.134	0.73	0.694	4.41
Overall	0.095	0.105	0.578	0.566	2.75

### Descriptive analysis

Descriptive analysis of the constructs revealed the mean score 3.40 (SD = 0.39) for Psychological Well-Being, showing that the participants had perceived themselves as moderately well psychologically. The distribution was approximately symmetrical (skewness = −0.08, SE = 0.18) with negligible deviation from normality (kurtosis = 0.35, SE = 0.35), indicating a bell-shaped curve. Job Content had a mean of 3.29 (SD = 0.44) and showed mild negative skewness (skewness = −0.54, SE = 0.18), implying a slight clustering of responses at the higher end of the scale. The kurtosis value of 1.24 (SE = 0.35) indicated a relatively peaked distribution. The mean score for Job Crafting was M = 3.76 (SD = 0.73), which is suggestive that participants generally reported a high level of proactive behavior in shaping their work roles. However, this variable displayed a strong negative skew (skewness = −1.71, SE = 0.18), and a high kurtosis (4.09, SE = 0.35), pointing to a distribution that is highly concentrated around the upper end with a pronounced peak—suggestive of non-normality. Work Identity had a mean of 3.46 (SD = 0.42), with near-symmetrical distribution (skewness = −0.12, SE = 0.18) and a modest kurtosis (0.39, SE = 0.35) (Table 5).

**Table 5:** Descriptive statistics, including mean ± standard deviation, skewness, and kurtosis for each major study variable.

	Mean ± Std. Dev	Skewness (± SE)	Kurtosis (± SE)
Psychological Well-being	3.4 ± 0.39	−0.08 ± 0.18	0.35 ± 0.35
Job Content	3.29 ± 0.44	−0.54 ± 0.18	1.24 ± 0.35
Job Crafting	3.76 ± 0.73	−1.71 ± 0.18	4.09 ± 0.35
Work Identity	3.46 ± 0.42	−0.12 ± 0.18	0.39 ± 0.35

### Correlational analysis

Results of the Pearson’s correlation analysis showed that Psychological Well-Being had a moderate positive correlation with Job Content (r = 0.292, p < 0.01) and more strongly correlated with Work Identity (r = 0.570, p < 0.01). Similarly, a moderately strong relationship was observed between Psychological Well-Being and Job Crafting (r = 0.488, p < 0.01). Job Content also had a significant positive association with Work Identity (r = 0.352, p < 0.01) and Job Crafting (r = 0.565, p < 0.01). In addition, Work Identity was positively correlated with Job Crafting (r = 0.448, p < 0.01), which is indicative that individuals who identify closely with their jobs are more inclined to engage in job crafting (Table 6).

**Table 6:** Correlation matrix showing interrelationships among Psychological Well-Being, Job Content, Work Identity, and Job Crafting, with 95% confidence intervals in parentheses. The significance levels are indicated as p < 0.05 (*), p < 0.01 (**), and p < 0.001 (***).

	Psychological Well-being	Job Content	Work Identity	Job Crafting
Psychological Well-being	1	0.292** (0.45 - 0.11)	0.570** (0.67 - 0.45)	0.488** (0.6 - 0.34)
Job Content	0.292** (0.45 - 0.11)	1	0.352** (0.49 - 0.19)	0.565** (0.69 - 0.4)
Work Identity	0.570** (0.67 - 0.45)	0.352** (0.49 - 0.19)	1	0.448** (0.57 - 0.3)
Job Crafting	0.488** (0.6 - 0.34)	0.565** (0.69 - 0.4)	0.448** (0.57 - 0.3)	1

### Regression analysis

In the model predicting Work Identity, Job Content emerged as a marginally significant predictor (β = 0.140, t = 1.850), while Job Crafting had a statistically significant effect (β = 0.210, t = 4.64, p < 0.001). Approximately, 21.5% of the variance of this model was contributed by Work Identity (R² = 0.215, F = 25.48, p < 0.001). In the model regarding Job Crafting, Job Content was a strong positive predictor (β = 0.780, t = 7.61, p < 0.001), while Work Identity also had a significant contribution (β = 0.490, t = 4.64, p < 0.001). Overall, these predictors had 39% of the contribution variance in Job Crafting (R² = 0.39, F = 59.56, p < 0.001). In the model used of prediction of Psychological Well-Being, the Work Identity was the strongest predictor variable (β = 0.440, t = 4.87, p < 0.001), followed by Job Crafting (β = 0.150, t = 2.52, p < 0.01). However, Job Content did not significantly predict Psychological Well-Being (β = 0.040, t = 0.430, p > 0.05). Overall, this model explained approximately 66.7% of the variance in Psychological Well-Being (R² = 0.667, F = 8.43, p < 0.001). Moreover, the demographic variables did not show significant contribution in any of the multivariable linear regression models (Table 7).

**Table 7:** Multiple regression analysis results showing standardized regression coefficients (β) and t-values for the predictive relationships among study constructs. The significance levels are indicated as p < 0.05 (*), p < 0.01 (**), and p < 0.001 (***).

	Work Identity		Job Crafting		Psychological Well-being	
	β	t	β	t	β	t
Job Content	0.140	1.850	0.780	7.61***	0.040	0.430
Job Crafting	0.210	4.64***	–	–	0.150	2.52**
Work Identity	–	–	0.490	4.64***	0.440	4.87***
R^2^	0.215		0.39		0.667379	
F	25.483216***		59.556834***		8.432***	

### Mediations analysis

**Fig 4:**
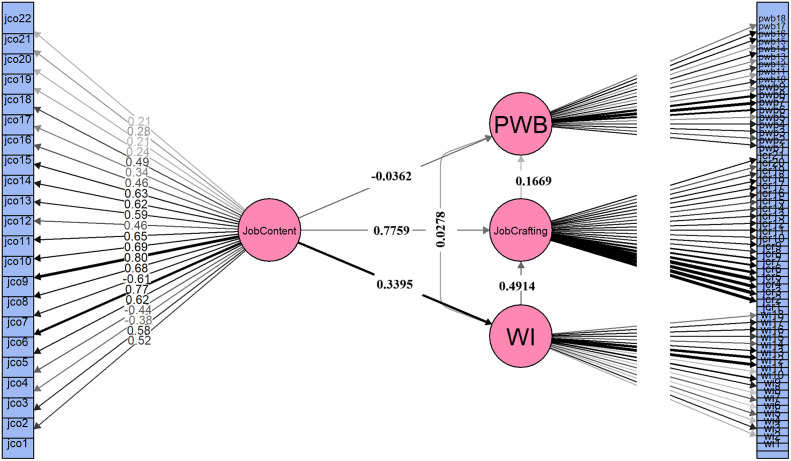
Structural Equation Model (SEM) depicting the hypothesized relationships among Job content, Work identity, Job crafting, and Psychological wellbeing. Standardized path coefficients are displayed along the arrows, with solid lines indicating significant paths (p < 0.05).

The results of mediation analysis revealed that the direct effect of Job Content on Psychological Well-Being was non-significant (β = −0.0362, p = 0.5627; 95% CI: −0.1594 to 0.0870) which indicates that Job Content alone does not have a statistically significant direct impact on Psychological Well-Being (Fig 4).

Two indirect pathways were found to be statistically significant. The first path was; Job Content → Work Identity → Psychological Well-Being, which yielded a standardized indirect effect of 0.1397 (p < 0.05; 95% CI: 0.0702 to 0.2139) and accounted for 53.56% of the total effect. This finding suggests that job content is associated with psychological well-being through work identity. On the other hand, the second pathway was; Job Content → Job Crafting → Psychological Well-Being, which also revealed a significant indirect effect (β = 0.1295, p < 0.05; 95% CI: 0.0544 to 0.2014) and represented 49.66% of the total effect. This result indicates the importance of proactive work design behaviors in transmitting the positive influence of job content on mental well-being. Additionally, the serial pathway Job Content → Work Identity → Job Crafting → Psychological Well-Being showed a smaller but statistically significant indirect effect (β = 0.0278, p < 0.05; 95% CI: 0.0080 to 0.0526), contributing 10.66% to the total effect. The total indirect effect was significant (β = 0.2970, p < 0.05; 95% CI: 0.1744 to 0.4141) and exceeded the direct effect (Table 8). The mediation models were also re-estimated with all demographic control variables (age, gender, experience, and sector) retained, and the results remained consistent in direction and significance, confirming the robustness of the findings. Given the cross-sectional design and model fit limitations, these mediation pathways should be interpreted as associative rather than causal.

**Table 8:** Mediation analysis results showing direct, indirect, and total effects of Job Content on Psychological Well-Being through Work Identity and Job Crafting, with bootstrapped confidence intervals.

Path	Effect Size	Boot LCI	Boot ULCI	p-value	Note
**Direct effects**					
JCo → WI	**0.3395**	0.1842	0.4781	<0.001	Significant
JCo → JCr	**0.7759**	0.5249	0.9966	<0.001	Significant
WI → JCr	**0.4914**	0.2818	0.6922	<0.001	Significant
JCo → PWB	**−0.0362**	−0.1594	0.087	0.5627	Nonsignificant
WI → PWB	**0.4114**	0.2892	0.5358	<0.001	Significant
JCr → PWB	**0.1669**	0.0793	0.242	<0.001	Significant
**Indirect effects**					
JCo → WI → PWB	**0.1397**	0.0715	0.214	<0.05	Parallel mediation
JCo → JCr → PWB	**0.1295**	0.0549	0.2026	<0.05	Parallel mediation
JCo → WI → JCr → PWB	**0.0278**	0.008	0.0533	<0.05	Serial mediation
**Total indirect effect**	**0.297**	0.174	0.4157	<0.05	
**Total effect**	**0.2608**	—	—	—	

## Discussion

The main purpose of our project was to assess the impact of job content on the psychological well-being of pharmacists nationwide, with a focus on the mediating roles of work identity and job crafting. Using serial and parallel mediation models, we studied the deeper understanding of how workplace structure and employee-driven behaviors interact to influence mental well-being. Our study was particularly focused on the Pakistani healthcare context, where pharmacists are working in high-pressure environments with limited professional resources and growing role expectations. However, the findings revealed that job content does not have direct influence on psychological well-being alone. Its effect was significantly mediated through two distinct pathways: via work identity and via job crafting, with both affecting independently and sequentially.

### The direct impact of job content on psychological well-being

The psychological well-being of the pharmacists in Pakistan is shaped by a complex interplay of workplace conditions and personal role engagement. In our study, while job content shows the workload and task structure but did not show a direct effect on well-being, its influence was only significant through the mediating roles of job crafting and work identity. Pharmacists facing demanding workloads may experience cognitive and emotional strain, leading to exhaustion and reduced mental resilience [70]. These effects are accompanied by limited opportunities for rest and recovery, and the frequent overlap between professional duties and familial responsibilities as the traditional roles and obligations further elevate stress levels (Alzoubi et al., 2024). The inability to directly manage or modify job demands may undermine well-being; however, those who actively reshape their roles and strongly identify with their work are better equipped to cope with these pressures. Our findings are in accordance with the prior studies which highlight how contextual stressors in South Asian healthcare environments uniquely burden professionals [71,72]. Hence, empowering pharmacists to engage in proactive role design and fostering a strong sense of professional identity may serve as critical buffers against the psychological impact of rigid or demanding job content. However, it is important to note that the cross-sectional nature of the data limits causal inference, and the observed mediation patterns reflect associations rather than directional effects in our study.

These findings suggest that structural job characteristics alone may not directly translate into improved well-being, especially in healthcare systems where autonomy is constrained by rigid hierarchies and procedural oversight [73]. In collectivist cultures like Pakistan’s, psychological well-being may depend less on structural autonomy and more on relational and identity-based factors such as belonging, recognition, and perceived purpose [74].

### Work identity as a mediator

The results of present study suggest the mediating role of work identity in the relationship between job content and psychological well-being among registered pharmacists in Pakistan. The results suggest that work identity was significantly associated with the relationship between job content and psychological well-being. Consistent with the mediation model, the indirect association through work identity was significant (β = 0.1397, p < 0.05). Our results are coinciding with previous studies which found that strong professional identity may serve as an important factor in enhancing resilience and overall psychological well-being of individuals [75]. It might be especially helpful to promote the work identity in Pakistan. A well-established work identity can provide pharmacists with a sense of purpose and belonging [76].

A strong professional identity appears to work as a psychological anchor that helps pharmacists to maintain stability even when external job demands to fluctuate [77]. Work identity can act as an inner motivational resource that sustains well-being despite limited organizational support, especially in healthcare professions, where moral purpose and social contribution are central to the sense of self [78].

### Job crafting as a mediator

The results of this project suggested the mediating role of job crafting in the relationship between job content and psychological well-being among the pharmacists in Pakistan. The results further suggest that job crafting was significantly associated with the relationship between job content and psychological well-being, with a significant indirect association observed through job crafting (β = 0.1295, p < 0.05). These results coincide with the Job Demands-Resources (JD-R) model, which proposes that employees proactively modify their job demands and resources to achieve better person-job fit [79]. Job crafting is an important strategy for achieving psychological well-being. Pharmacists can reduce their jobs associated pressures, improve their engagement and happiness by transforming their work roles. In addition to helping individual pharmacists, this proactive conduct also increases the effectiveness and resilience of the organization [80].

### Sequential mediation of Work Identity and Job crafting

The current study gives evidence supporting the sequential mediating roles of work identity and job crafting among relationship between job content and psychological well-being among registered pharmacists’ nationwide. A smaller but significant sequential indirect association was also observed through work identity and job crafting (β = 0.0278, p < 0.05). These findings are consistent with the Job Demands-Resources (JD-R) model, which describe that job resources can support personal resources, leading to enhanced well-being [81].

### The job culture context of Pakistan

Significant job structural changes are necessary to address pharmacists’ psychological well-being, particularly in countries like Pakistan with systemic resource constraints [82]. Encouraging Job crafting and building a strong sense of “who we are” at work may help maintaining good mental health [83]. Future strategies should also consider integrating mindfulness-based interventions into pharmacist training programs, as these can equip individuals with effective stress management tools without imposing additional burdens on managerial oversight. Many studies have pointed out that these interventions can make people happier at work, less stressed and enjoy greater life satisfaction [84,85]. Meanwhile, the perceived organizational support that is manifested through adequate infrastructure, supportive leadership, and positive work environments can enhance the effects of these individual efforts. Studies have demonstrated that better organizational support is also linked to reduced stress levels and better mental health [86]. Therefore, a combined approach of using individual-level strategies such as job crafting and mindfulness with organizational-level support systems could prove the most effective in provoking resilience and promoting mental well-being among pharmacists.

Altogether, the results suggest that interventions aiming to enhance pharmacists’ psychological well-being should go beyond traditional job redesign and workload management. Strategies that strengthen personal meaning, encourage reflective practice, and provide space for professional autonomy could yield more sustainable outcomes [87].

### Limitations

The selection bias observed in cross-sectional studies limits causal inference among the examined variables. Additionally, potential confounding factors such as personality traits, perceived organizational support, and work–life balance could not be fully controlled and may have affected the observed relationships. Additionally, as all variables were assessed using self-reported measures collected at a single time point, the findings may be subject to common method bias. Furthermore, the exclusion of individuals with existing psychological conditions and the focus on licensed pharmacists may limit the generalizability of the findings to broader or more diverse populations. Importantly, the Psychological Well-Being scale demonstrated marginal internal consistency (α = 0.66) and low convergent validity (AVE = 0.35), and the overall structural model showed poor fit indices (i.e., CFI and TLI). These limitations in AVE and item loadings may affect convergent validity and should be considered when interpreting the findings. Similar psychometric challenges have been reported in prior studies using Ryff’s Psychological Well-Being scales in non-original and cross-cultural contexts, where cultural interpretation of items, method effects, and multidimensionality have been shown to attenuate convergent validity and compromise overall model fit [64–66,88,89]. These model-fit limitations may reduce confidence in the precision of parameter estimates and restrict the strength of directional conclusions. Therefore, the findings should be interpreted as exploratory rather than confirmatory, and future research should employ culturally validated well-being instruments and longitudinal designs to strengthen inference.

## Conclusion

The study suggests that the psychological well-being of pharmacists in Pakistan is influenced by their work identity and job crafting behaviors, rather than job content alone. Although psychological well-being was not directly associated with job content, it appeared to be indirectly linked through work identity and job crafting. However, these findings should be interpreted with caution given the limitations in model fit and the exploratory nature of the analysis.
